# Sex, Sin, and Science: A History of Syphilis in America

**DOI:** 10.3201/eid1506.090308

**Published:** 2009-06

**Authors:** Thomas A. Peterman

**Affiliations:** Centers for Disease Control and Prevention, Atlanta, Georgia, USA

**Keywords:** Bacteria, syphilis, history, social diseases, sexually transmitted infections, sin, book review

At of end his book, John Parascandola writes, “It is my hope that the reader ... acquired a broader appreciation of disease as a social as well as a medical construct and of the way in which social and cultural factors influence our understanding of and reaction to any given disease.” The social context of syphilis is nicely summarized by the title, Sex, Sin, and Science: A History of Syphilis in America. Because syphilis is sexually transmitted, it is often considered as a moral issue, and thus people who have syphilis have sinned. This perception is both absurd and insightful.

Parascandola’s history of syphilis is compelling from the beginning. “Because many believed that the disease first made its appearance in the French troops besieging Naples, it was often called (especially by the Italians) *morbus gallicus* (‘French disease’) ... The French, on the other hand, preferred to call it the ‘Neapolitan disease’ blaming it on the city of Naples.”

Syphilis was so sinful that it could not be discussed by name. “This continued hesitancy to discuss sexual matters is reflected in the terminology used in newspapers and other public media of the early twentieth century.” “Social evil” meant prostitution. Syphilis and gonorrhea were “social diseases,” and the effort to combat them was the “social hygiene” movement. We learn that in 1911 California became the first state to require physicians to report cases of venereal disease and that, to assure confidentiality, reporting was done by number rather than by name.

Some of those involved in the social hygiene movement were more interested in preventing sex than in preventing disease. A Public Health Service (PHS) advisory committee recommended changing an educational film so that “‘some attention be given to the influence of moral standards on the spread of disease’ because if no reference was made to moral issues, it might appear to some that the PHS was ‘condoning sexual promiscuity.’”

There was fear that penicillin might offer “complete freedom to indulge in licentiousness...” or “if extramarital sex did not lead to significant illness, only a ‘few intangibles of the spirit’ would remain to guide people into moral paths.” Parascandola notes, “Social hygienists had always been at least as interested in moral as in health issues, and so their fight would not end with the defeat of venereal disease.”

Parascandola’s book informs readers that in 1953, “the Eisenhower administration proposed eliminating the PHS venereal disease program because its job was essentially done.” But syphilis came back, along with gonorrhea, herpes, chlamydia, and AIDS.

There is considerable scientific evidence that HIV causes AIDS. Nonetheless, just as with syphilis, others think the cause is sin. Absurd. Yet the social construct Parascandola describes remains so pervasive that it continues to affect us all.

